# Functional Biomaterials and Digital Technologies in Dentistry: From Bench to Bedside

**DOI:** 10.3390/jfb15040107

**Published:** 2024-04-17

**Authors:** Ping Li, Guojiang Wan, Shulan Xu, An Li

**Affiliations:** 1 Department of Prosthodontics, School and Hospital of Stomatology, Guangzhou Medical University, Guangzhou 510182, China; 2 Key Laboratory of Advanced Technologies of Materials (Ministry of Education), School of Materials Science and Engineering, Southwest Jiaotong University, Chengdu 611756, China; guojiang.wan@home.swjtu.edu.cn; 3 Center of Oral Implantology, Stomatological Hospital, School of Stomatology, Southern Medical University, Guangzhou 510280, China; xushulan_672588@smu.edu.cn; 4 Department of Periodontology, Stomatological Hospital, School of Stomatology, Southern Medical University, Guangzhou 510280, China

This Special Issue, “Functional Biomaterials and Digital Technologies in Dentistry: From Bench to Bedside”, highlights the integration of advanced materials science and digital technologies in dental and maxillofacial applications. We sincerely appreciate the Journal of Functional Biomaterials and the committed researchers for making this Special Issue possible through their contributions.

This Special Issue consists of 11 articles that include 6 original research studies (contributions 1–6) and 5 reviews (contributions 7–11) focusing on a broad spectrum of functional biomaterials and digital technologies in the field of dental applications. The research presented encompasses various applications, ranging from metallic and polymer biomaterials to organic and composite biomimetic materials. Additionally, this Special Issue emphasizes advancements in digital technologies, particularly in computer-aided imaging (CAI), computer-aided design (CAD), and computer-aided manufacturing (CAM).

## 1. Why We Focus on the Topic of “Functional Biomaterials and Digital Technologies in Dentistry: From Bench to Bedside”

The thematic focus of “Functional Biomaterials and Digital Technologies in Dentistry: From Bench to Bedside” is deliberate and dictated by the revolutionary developments in the field. Dentistry is currently at the cusp of a transformative epoch, fueled by material science, engineering, and computer science. These disciplines are pivotal in sculpting the future of dental science. Functional biomaterials, recognized for their enhanced biocompatibility, robust mechanical properties, and tailored biofunctionality, are paving the way for groundbreaking developments in oral and maxillofacial treatments. Furthermore, digital technologies have catalyzed a revolutionary shift in various healthcare sectors, including dentistry. Sophisticated methodologies such as CAD/CAM, 3D printing, bioprinting, and dental virtual patients have emerged as powerful tools, substantially improving the accuracy of procedures, patient experiences, and efficiency of dental care.

## 2. Special Issue of “Functional Biomaterials and Digital Technologies in Dentistry: From Bench to Bedside”

### 2.1. Functional Biomaterials in Dentistry

Within the field of dentistry, a diverse range of biomaterials—including metals, polymers, ceramics, and composites—is commonly utilized in creating dental implants, dental resin, osteosynthesis implants, and scaffolds for both bone and soft tissue regeneration [1,2,3]. Ongoing developments and investigations of new materials are propelled by their intrinsic biofunctional properties, which hold the promise to enhance clinical outcomes significantly [4,5].

- Surface modification of dental implant abutment: In this Special Issue, Wen et al. (contribution 5) investigated the impact of UVC (100–280 nm) pre-treatment on human gingival fibroblasts (HGFs) and *Porphyromonas gingivalis* (*P. gingivalis*) interactions with Ti-based implant surfaces. The results indicated that UVC pre-treatment improves HGF adhesion and proliferation while diminishing *P. gingivalis* colonization on smooth Ti substrates relative to untreated controls.
- Optimization of dental resin: Within this Special Issue, Bourgi et al. (contribution 10) investigated the influence of varying temperatures of warm air on solvent evaporation from dental adhesives and its subsequent effect on the bond strength of resin-based materials to dental and nondental substrates. The systematic review of in vitro studies identified that the optimal temperature range for a warm air stream to promote solvent removal and enhance adhesion to dentin is between 50 and 60 °C.
- Biodegradable metals for osteosynthesis implant: Zinc (Zn)-based biodegradable materials are emerging for use in osteosynthesis implants, despite inconsistencies between in vitro and in vivo biocompatibility [6,7]. In this Special Issue, Liu et al. (contribution 9) examined the cytotoxic potential of Zn and its alloys, revealing no harmful effects under specific conditions. Of note, the systematic review highlighted significant inconsistencies in cytotoxicity testing approaches.
- Scaffolds for bone tissue regeneration: Bone tissue regeneration employs a synergistic combination of biocompatible scaffolds, specialized cells, and growth factors, meticulously engineered to support and accelerate new bone formation, meeting both mechanical and biological needs [8]. Research by Yang et al. (contribution 6) highlighted the osteoinductive potential of RADA16 nanofiber scaffold hydrogel-wrapped concentrated growth factors for treating alveolar bone loss, while Huang et al. (contribution 8) reviewed the application of stem cell-derived extracellular vesicles in periodontal osteogenesis therapy, focusing on the RANKL/RANK/OPG pathway’s role.
- Scaffolds for soft tissue regeneration: Soft tissue regeneration aims to repair or replace damaged tissues such as muscle and skin through biocompatible scaffolds, growth factors, and cells, ensuring functional restoration and integration. In this Special Issue, Li et al. (contribution 2)’s research depicts an enhancement of the bioactivity and biocompatibility of mesoporous silica nanospheres with liquid crystal, demonstrating the potential of the hydrogel for effective soft tissue regeneration. Concurrently, Dong et al. (contribution 1) probe the cytotoxic effects of black phosphorus nanosheets on vascular endothelial cells for tissue regeneration, uncovering a mechanism driven by excessive reactive oxygen species production and mitochondrial dysfunction leading to cell apoptosis.

### 2.2. Digital Technologies in Dentistry

Digital dentistry revolutionizes patient care by enhancing diagnostics with CAI and CBCT, and improving restorations via CAD/CAM for quicker, more precise outcomes [9]. Integrated digital workflows boost treatment predictability and efficiency, while 3D printing, artificial intelligence (AI), and robotic surgery offer customized treatments, significantly enhancing patient outcomes and experiences.

- Computer-aided imaging

CAI technology in dentistry enhances diagnosis and treatment planning through advanced digital imaging, such as intraoral scans and CBCT [10,11]. This technology provides precise visual data for better patient outcomes. Saravi et al.’s study evaluated the use of computerized optical impression making (COIM) in partially edentulous jaws, highlighting potential deviations in prosthetics involving mucosal tissues. Yuan et al. reported on the successful integration of stomatognathic data from various devices for creating dynamic virtual patients, showcasing the advancements and considerations in CAI applications (contribution 11).

- Computer-aided design and manufacturing

CAD/CAM technology in dentistry represents a transformative approach to dental restoration, offering a streamlined process from design to production. In this issue, Wang et al. (contribution 4) investigated the adhesion characteristics of a 3D-printed polyetheretherketone (PEEK) material. Their findings suggested that surface texture alone does not exclusively dictate the adhesion qualities of additively produced PEEK. Additionally, Chen et al. (contribution 7) provided a comprehensive review of the evolution of obturator fabrication for oronasal fistulas following cleft palate repair, tracing its progression from manual craft to the implementation of advanced digital methodologies.

## 3. Summary

Through curating this Special Issue, we place a spotlight on the fusion of functional biomaterials and digital advancements as a decisive force propelling dental care forwards. Offering treatments that are more personalized, efficient, and effective, these technologies align with the broader transition towards precision medicine. The customization of treatments to meet individual needs, utilizing biomaterials with superior biocompatibility and mechanical properties, is expected to improve health outcomes significantly. Additionally, the incorporation of digital technologies in dentistry is set to transform the intricacies of diagnostics and treatment, making dental care more accessible and cost-effective. This Special Issue not only provides an overview of current research and development in the field but also shines a light on potential shifts in dental care. Looking forwards, the intersection of functional biomaterials and digital technology is poised to redefine the future of dental and maxillofacial treatments, ensuring advanced care for patients worldwide.

## Data Availability

The data presented in this paper are available upon request from the corresponding author.
